# Perirenal fat thickness as a risk factor for postoperative complications in elective colorectal cancer surgery

**DOI:** 10.1097/MD.0000000000034072

**Published:** 2023-06-23

**Authors:** Mehmet Reşit Sönmez, İsa Caner Aydin, Gülşah Biçer, Nuri Havan, Ahmet Orhan Sunar, Serkan Ademoğlu, Mehmet Ömer Özduman, Mürşit Dinçer, Erdal Polat, Mustafa Duman

**Affiliations:** a Gastroenterological Surgery Clinic, University of Health Sciences, Ministry of Health, KartalKosuyolu High Specialization Training and Research Hospital, Istanbul, Turkey; b Department of Radiology, University of Health Sciences, Ministry of Health, KartalKosuyolu High Specialization Training and Research Hospital, Istanbul, Turkey.

**Keywords:** colorectal cancer, perirenal fat thickness, postoperative complications, prognosis, visceral obesity

## Abstract

Visceral obesity is an important factor that increases the risk of complications after colorectal cancer surgery. As calculating visceral fat is difficult and time-consuming, more practical fat measurements that are not time-consuming have been introduced. This study aimed to investigate the effects of perirenal fat thickness on postoperative complications and prognosis in patients undergoing surgery for colorectal cancer. Perirenal fat thickness was measured from the dorsal aspect of the left kidney on preoperative computerized tomography of patients who underwent surgery for colorectal cancer. The effects of perirenal fat thickness on postoperative complications were investigated. Diagnostic test performance was examined using the Roc Curve test to determine the cutoff value for the perirenal fat thickness values according to the complication findings of the patients. The cutoff value of perirenal fat thickness was found to be above 25.1, according to the presence of complications in the patients. Those with a perirenal fat thickness greater than 25.1 mm were considered to have high perirenal fat thickness values, and those with a low perirenal fat thickness value were considered low. Multivariate analysis revealed that increased perirenal fat thickness is an independent risk factor for postoperative complications. We believe that perirenal fat thickness measurement, as an indicator of visceral fat volume, can be used to identify patients at high risk of developing complications after colorectal cancer surgery. This may change the disease management and affect the patient information process.

## 1. Introduction

Complications after colorectal cancer (CRC) surgery are common.^[1]^ Complications delay patient recovery, prolong hospital stay, and impair the quality of life. This may delay adjuvant therapy.^[2]^ Preoperative risk assessment aims to prevent complications.^[3]^ Risk assessment allows early identification of the need for care and reduces morbidity.^[4]^

Studies have shown that visceral fat volume (VFV) rather than total body fat is associated with a greater increase in postoperative complications.^[1,5–7]^ It has been reported that visceral obesity also leads to poor oncological outcomes in CRC patients.^[2,8,9]^

Preoperative computed tomography (CT) can be used to measure the VFV at no additional cost. Visceral fat analysis using CT uses the currently acquired CT images of the patients. The perirenal fat area has been reported to be an indirect measurement that correlates with VFV and is associated with complications after colorectal surgery.^[1,10]^ However, these fat area measurements require special imaging software and are time consuming. Recent research has sought more practical methods to assess obesity using CT imaging.

An easier method representative of visceral fat is the measurement of perirenal fat thickness (PFT). In the literature review, the number of studies on this subject is rare and they offer contradictory results.^[1,8,10]^

This study aimed to investigate the effects of perirenal fat thickness on postoperative complications and prognosis in patients undergoing surgery for colorectal cancer.

## 2. Material and method

Preoperative CT scans, archived files, and computer records of 202 patients who underwent surgery for stage I to IV colorectal carcinoma at the Gastroenterology Surgery Clinic of the Kartal Kosuyolu High Specialization Training and Research Hospital between July 2017 and December 2021 were retrospectively reviewed.

The pathological data of the patients were recorded. The Death Notification System was used, or patients were telephoned to determine overall survival.

In addition, age, sex, T stage, lymphovascular invasion (LVI), and grade of the patients were noted, and their prognostic significance was evaluated using statistical analysis.

The inclusion criteria in this study are as follows:

1. Elective and curative surgery,
2. Histologically confirmed diagnosis of carcinoma,
3. Full clinicopathological features and follow-up data.

The exclusion criteria are as follows:

1. Palliative or emergency surgery,
2. Presence of chronic liver disease, acute or chronic inflammatory disease, infection, or any systemic disease.
3. Presence of unresectable tumor.

### 2.1. Demographic and clinical data

Information about the patient’s age, sex, body mass index (BMI), tumor location, operation time, and postoperative complications was obtained from hospital records.

Age, sex, and BMI were considered patient-related factors. Tumor-related variables included the tumor location, tumor size, pathologic T-stage, pathologic N-stage, M, LVI, and perineural invasion.

Grade II or higher postoperative complications were included according to the Clavien-Dindo classification. Complications were categorized as anastomotic leakage or other. Routine intraoperative air leak test or routine postoperative CT examination haven’t been done to determine anostomotic leakage. In cases of CRP, WBC elevation, presence of fever, and clinical worsening, CT with rectal contrast was performed to reveal leakage. Other complications include intra-abdominal infections, wound infections, and pneumonia.

Pathologic T-stage, pathologic N-stage, and M classification were performed according to the 8th edition of TNM classification of the Union for International Cancer Control – American Joint Committee on Cancer (UICC-AJCC). Patients with BMI < 25 kg/m^2^ were classified as normal weight and BMI ≥ 25 kg/m^2^ as overweight.

### 2.2. Measurement of PFT

**Figure 1.**
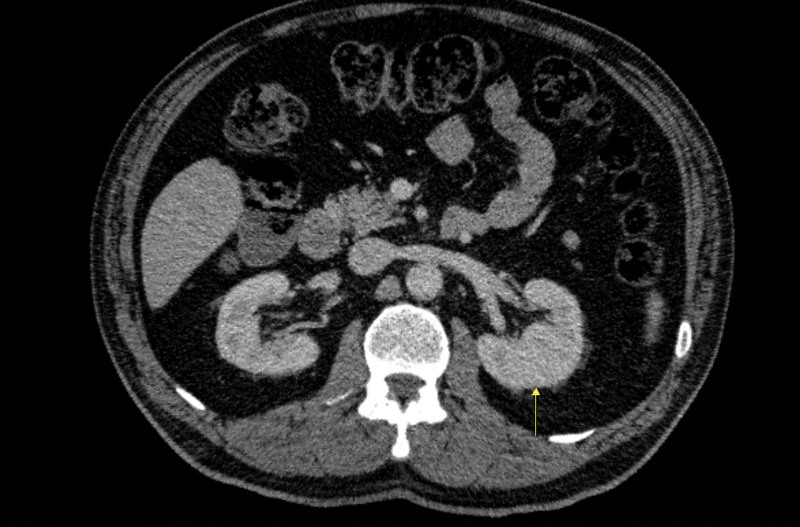
Measurement of perirenal fat thickness.

PFT distance was measured from the anterior surface of the Musculus Quadratus Lumborum to the dorsal edge of the left kidney pole at the exit level of the renal vein in the axial CT section (Fig. 1). Diagnostic test performance was examined using the Roc Curve test to determine the cutoff value for the PFT values according to the complication findings of the patients. The cutoff value of PFT was found to be above 25.1, according to the presence of complications in the patients. Those with a perirenal fat thickness greater than 25.1 mm were considered to have high PFT values, and those with a low PFT value were considered low.

### 2.3. Statistical evaluation

Data were analyzed using the Statistical Package for the Social Sciences 25.0 application. Numbers and percentages were used to represent categorical measurements, whereas mean and standard deviation were used to summarize continuous measures (median and minimum-maximum where appropriate). Categorical expressions were compared using chi-square and Fisher’s exact tests. The Kolmogorov–Smirnov test was used to determine if the study parameters had a normal distribution. The independent Student *t* test was used for normally distributed parameters; the Mann–Whitney *U* test was used for parameters that did not show a normal distribution to the patients’ complications. The sensitivity and specificity of the PTF values were calculated according to patient complications. The cutoff value was established by examining the area under the ROC curve (AUC). Kaplan–Meier and log-rank tests were used to assess patient survival. To investigate the variables influencing morbidity, a logistic regression test was used in the univariate analysis and a multiple logistic regression test in the multivariate analysis. For each test, the statistical significance level was set at *P* < .05.

### 2.4. Ethical consideration

KartalKosuyolu High Specialization Training and Research Hospital Scientific Ethics Committee approved the data collection (Decision No: 2022/9/596 Date: 10.05.2022).

## 3. Results

A total of 202 patients who had undergone colorectal cancer surgery were included in this study. The mean age of the patients was 60.6 ± 13.3 years, BMI was 26.1 ± 5.2, and 128 (63.4%) were male. Leakage was present in 23 patients (11.4%), other complications in 48 patients (23.8%), and total complications in 66 patients (32.7%). Tumor locations were as follows: 26 cecum (12.9%), 31 ascending colon (15.3%), 6 transverse colon (3%), 13 descending colon (6.4%), 65 sigmoid colon (32.2%), and 61 rectum (30.2%). Leakage rates were 13 of 61 (21%) for the rectum tumors and 10 of 141 (7%) for the colon tumors. Leakage rate is significantly higher in the rectum tumor group than the colon tumor group (p:0.0173).

**Figure 2.**
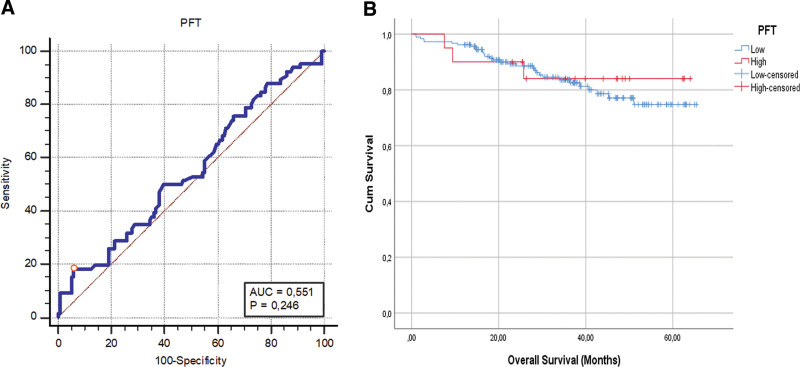
A. Examination of PFT value with Roc Curve test according to the presence of complications. B. Comparison of the mean survival among the PFT groups. PFT = perirenal fat thickness.

The diagnostic test performance with the Roc Curve test (Fig. 2) was analyzed to determine the cutoff value for the PFT values according to the complication findings of the patients. According to the examination, the cutoff value of the PFT value was 25.1 mm higher than the presence of complications in the patients; It was determined that the area under the AUC curve was 55.1%, its sensitive was 18.18%, and its specificity was 94.12%. The cutoff values obtained were not statistically significant (*P* > .05). This value was not statistically significant because of the small number of patients included in the study. If there were enough patients, this would have affected the number of patients staying above this value. Therefore, the relationship between this value and complications may be significant.

Anastomotic leakage and total incidence of complications were higher in patients with high PFT values. The mean age and BMI of the patients with high PFT values were found to be high. The presence of LVI was low in the patients with high PFT values. No significant differences were found in other parameters between the groups (Table 1).

**Table 1:** Differences between demographic findings and PFT groups.

	PFT low (n = 182)	PFT high (n = 20)	*P* †
	n (%)	n (%)	
Gender			
Male	112 (61.5)	16 (80)	.104
Female	70 (38.5)	4 (20)	
Tumor location			
Colon	128(70.3)	13 (65)	.541
Rectum	54 (29.7)	7 (35)	
T			
1	22 (12.1)	4 (20)	.602
2	23 (12.6)	3 (15)	
3	114 (62.6)	12 (60)	
4	23 (12.6)	1 (5)	
N			
0	108 (59.3)	14 (70)	.111
1	41 (22.5)	6 (30)	
2	33 (18.1)	-	
M			
0	166 (91.2)	18 (90)	.914
1	16(8.7)	2 (10)	
LVI	66 (36.3)	3 (15)	.044*
PNI	57 (31.3)	5 (25)	.561
Neoadjuvant chemotherapy	45 (24.7)	5 (25)	.978
Laparoscopic surgery	45 (24.7)	3 (15)	.332
Anastomotic leak	17 (9.3)	6 (30)	**.006** *
Other complication	42 (23.1)	6 (30)	.490
Total complication	54 (29.7)	12 (60)	**.006** *
Mortality	31 (17)	3 (15)	.818
	**Mean ± SD**	**Mean ± SD**	***P*** ‡
Age	59.9 ± 13.4	66.8 ± 10.4	**.031** *
BMI	25.6 ± 5.0	30.3 ± 4.5	**<.001** **
Total number of lymph nodes	23.4 ± 10.8	20.3 ± 9.5	.165
Surgery time, h	4.81 ± 2.7	5.30 ± 2.9	.444
Hospital stay, d	10.6 ± 8.9	13.9 ± 12.9	.128

Bold values denote statistical significance at the *P* <.05 level.

LVI = lymphovascular invasion, M = metastasis, N = lymph node, PFT = perirenal fat thickness, PNI = perineural invasion, T = tumor.

* *P* < .05.

** *P* < .001.

† Chi-square and Fisher exact.

‡ Mann–Whitney *U* test.

While the mean survival time of the patients was found to be 56.0 ± 1.45 months; It was determined that the mean survival times of patients with low and high PFT values were similar (*P* = .667) (Fig. 2).

High PFT, administration of neoadjuvant chemotherapy, laparoscopic surgery, mortality, operative time, and length of hospital stay were higher in patients with complications (Table 2).

**Table 2:** Differences between demographic findings and complication groups.

	No complication (n = 136)	Complication (n = 66)	*P* †
	n (%)	n (%)	
Gender			
Male	82 (60.3)	46 (69.7)	.193
Female	54 (39.7)	20 (30.3)	
Tumor location			
Colon	100 (73.5)	43 (65)	.443
Rectum	36 (26.5)	23 (35)	
T			
1	19 (14)	7 (10.6)	.783
2	19 (14)	7 (10.6)	
3	82 (60.3)	44 (66.7)	
4	16 (11.8)	8 (12.1)	
N			
0	79 (58.1)	43 (65.2)	.148
1	37 (27.2)	10 (15.2)	
2	20 (14.7)	13 (19.7)	
M			
0	125 (91.9)	59 (89.4)	.342
1	11 (8.1)	10 (10.6)	
LVI	51 (37.5)	18 (27.3)	.151
PNI	49 (36)	13 (19.7)	**.018** *
Neoadjuvant chemotherapy	28 (20.6)	22 (33.3)	**.049** *
Laparoscopic surgery	39 (28.7)	9 (13.6)	**.018** *
PFT			
<25.1 (Low)	128 (94.1)	54 (81.8)	**.006** **
>25.1 (High)	8 (5.9)	12 (18.2)	
Mortality	17 (12.5)	17 (25.8)	**.018** *
	**Mean ± SD**	**Mean ± SD**	***P*** ‡
Age	60.4 ± 12.9	61.1 ± 14.2	.624
	60.5 (63)	63 (56)	
BMI	26.1 ± 4.8	26.2 ± 5.8	.752
	25.25 (21.9)	25 (33.8)	
Total number of lymph nodes	22.7 ± 11.1	23.9 ± 9.8	.332
	20 (60)	22 (57)	
Surgery time, h	4.5 ± 2.7	5.6 ± 2.6	**.009** **
	5 (9)	7 (9)	
Hospital stay, d	6.8 ± 1.5	19.3 ± 12.8	**<.001** **
	7 (11)	15 (80)	

Bold values denote statistical significance at the *P* < 0.05 level.

LVI = lymphovascular invasion, M = metastasis, N = lymph node, PFT = perirenal fat thickness, PNI = perineural invasion, T = tumor.

* *P* < .05.

** *P* < .001.

† Chi-square and Fisher exact.

‡ Mann–Whitney *U* test.

According to Univariate analysis, high PFT was 3.56 times (OR: 3.556), perineural invasion presence was 0.436 times (OR: 0.436), laparoscopic surgery 0.39 times (QR: 0.393), and operation time 1.16 times (OR: 1.166), which was found to increase the development of complications. Parameters that were found to be significant in the univariate analysis were included in the multivariate analysis. According to the results of multivariate analysis, a high PFT increased complications 31.34 times (OR: 31.341) (Table 3).

**Table 3:** Univariable and multivariable analysis of factors affecting postoperative morbidity.

	Univariate		Multivariate	
	Hazard ratio (95% CI)	*P*	Hazard ratio (95% CI)	*P*
Age (yr)				
<75	1000			
≥75	0.818 (0.354–1892)	.639		
Gender				
Male	1000			
Female	0.6630 (0.353–1236)	.195		
PFT				
<25.1 (Low)	1000		1000	
>25.1 (High)	3556 (1376–9189)	**.009** **	31.341 (2539–.386.92)	**.007** **
Neoadjuvant chemotherapy	1929 (0.997–3729)	.051		
T				
1	1000	.784		
2	1000 (0.294–3406)	1000		
3	1456 (0.568–3731)	.433		
4	1357 (0.404–4565)	.622		
N				
0	1000	.156		
1	0.497 (0.225–1095)	.083		
2	1194 (0.541–2634)	.660		
M				
0	1000	.490		
1	0.286 (0.037–2333)	.233		
LVI (1)	0.625 (0.328–1189)	.152		
PNI (1)	0.436 (0.216–0.877)	**.020** *	0.084 (0.007–1018)	.052
Laparoscopic surgery	0.393 (0.177–0.870)	**.021** *	1883 (0.301–11.180)	.511
BMI	1007 (0.951–1066)	.811		
Total number of lymph nodes	1011 (0.983–1039)	.446		
Surgery time, h	1166 (1038–1310)	**.010** *	1042 (0.753–1442)	.804

Bold values denote statistical significance at the *P* < 0.05 level.

Univariate: Logistic regression, Multivariate: Multiple logistic regression.

LVI = lymphovascular invasion, M = metastasis, N = lymph node, PNI = perineural invasion, T = tumor.

* *P* < .05.

** *P* < .001.

## 4. Discussion

Despite decreased complications and hospital stays after colorectal cancer surgery, postoperative complications remain an important problem.^[11–13]^ It is vital to preoperatively identify high-risk patients and develop preventive measures against complications.

We examined the effect of PFT as an indicator of VFV on postoperative complications and prognosis in patients undergoing CRC surgery. We found the cutoff value of PFT above 25.1 mm and that the increase in PTF was associated with an increase in complications after CRC surgery but did not have a significant effect on mortality and overall survival. We conclude that a high PFT could be used to identify patients at a high risk of developing complications after CRC surgery and to make changes in management.

The BMI is widely used in the diagnosis of obesity. Many studies have reported that high BMI causes increased postoperative complications in patients.^[14–16]^ However, other studies have reported that there can still be an increase in complications in patients with a normal BMI owing to increased visceral fat.^[8,17–19]^ BMI includes both visceral and subcutaneous fat but cannot determine the ratio of these components.

Serious complications such as anastomotic leakage are thought to be due to difficult dissection because of abundant visceral fat and a narrow operative field. In males, a narrow pelvis and more visceral fat may negatively affect the postoperative outcome of CRC surgery. This may explain the greater technical difficulties and worse oncological outcomes of performing surgery in male patients.^[16,20]^ Although anastomotic leakage was significantly higher in the group with a high PFT as a manifestation of visceral obesity in our study, there was no difference between sexes.

Leakage rates were 13 of 61 (21%) for the rectum tumors and 10 of 141 (7%) for the colon tumors. Leakage rate is significantly higher in the rectum tumor group than the colon tumor group (*P*: 0.0173). The higher rate of leakage in rectal tumor surgery is consistent with our clinical experience and expectations.

Tsujinaka et al^[8]^ reported that VFV is a more efficient predictor of surgical complications. Quirt et al^[17]^ showed that visceral obesity increases postoperative complications in patients with normal BMI. In another study conducted in patients who underwent laparoscopic rectal resection, visceral obesity was not associated with the development of anastomotic leakage or other complications but was associated with an increased risk of conversion to open surgery.^[21]^ Jung et al^[1]^ showed that perirenal fat surface area has a higher predictive value for postoperative outcomes in colorectal surgery than BMI and waist-to-hip ratio. der Hagopian et al^[22]^ reported in their study that perirenal fat surface area, which is an indirect marker of visceral obesity, is associated with complications and can serve as a useful tool for preoperative risk assessment.

Measurement of the volume and area of visceral fat is complex, requires specialized software, and does not appear to be practical for clinical use. Instead, PFT is an easier and more practical method for visceral fat measurements.^[10]^

Eto et al^[10]^ reported that the cutoff value of PFT as an indicator of visceral fat was > 11.2 mm in patients who underwent laparoscopic distal gastrectomy for gastric cancer. They reported that a high PFT value is associated with an increased complication rate. In the literature search, no other study has reported a cutoff value for PFT.

We found a cutoff value of PFT > 25.1 mm in patients who underwent CRC surgery and a significant increase in complications in patients whose PFT values were higher than the cutoff value in our study. However, we did not find a significant relationship between BMI and complication rates.

Eckberg et al^[23]^ reported no relationship between PFT and overall survival in patients undergoing elective colon cancer surgery. Park et al^[24]^ reported that increased visceral obesity prolongs overall survival by preventing lymph node metastasis in colorectal cancer.

We did not find a significant difference between the PFT groups in terms of mortality and overall survival in our study. However, the rate of lymphovascular invasion was low in patients with high PFT. This finding supports the hypothesis that visceral obesity prevents lymph node metastasis, as reported by Park et al.

Many reliable prediction systems are available for estimating surgical risk for poor postoperative outcomes, including the POSSUM,^[25]^ modified POSSUM score,^[26]^ and Estimation of Physiological Ability and Surgical Stress (E-PASS) scoring system.^[27]^ As these estimation methods use many preoperative and intraoperative variables, they are time consuming and difficult to use. Estimation based on PFT measurement is based only on CT images, which can be routinely measured preoperatively. PFT measurement is an easier and more practical method to use in clinical practice.

Although obesity is classified as one of the non-adjustable risk factors for anastomotic leakage in some articles, it is considered as a modifiable factor in some studies. There is generally no preoperative risk-reducing precaution; because there is little time before surgery.^[28,29]^ Patients who are planned for neoadjuvant treatment can be included in the diet program during this period, if PFT is high. Intraoperative air leak test or colonoscopy control can be planned in patients with high PFT. The chance of opening a proximal diverting ostomy should be considered.^[28]^ In the postoperative period, suspicion for leakage should be kept at a high level.

A limitation of our study is that it was a retrospective, single-center study. We believe that prospective multicenter studies are needed.

In conclusion, this study showed that PFT is a simple and useful predictive measure for postoperative complications in patients with colorectal cancer. We believe that PFT measurement, as an indicator of VFV, can be used to identify patients at high risk of developing complications after colorectal surgery. This may change the disease management and affect the patient information process.

## Author contributions

**Conceptualization:** Mehmet Reşit Sönmez, İsa Caner Aydin, Gülşah Biçer, Mürşit Dinçer, Mustafa Duman.

**Data curation:** Mehmet Reşit Sönmez, İsa Caner Aydin, Gülşah Biçer, Nuri Havan, Ahmet Orhan Sunar, Mehmet Ömer Özduman, Mürşit Dinçer.

**Formal analysis:** Mehmet Reşit Sönmez, Gülşah Biçer, Nuri Havan, Mürşit Dinçer, Mustafa Duman.

**Funding acquisition:** Mehmet Reşit Sönmez.

**Investigation:** Mehmet Reşit Sönmez, İsa Caner Aydin, Gülşah Biçer, Serkan Ademoğlu.

**Methodology:** Mehmet Reşit Sönmez, İsa Caner Aydin, Gülşah Biçer, Serkan Ademoğlu, Mehmet Ömer Özduman.

**Project administration:** Mehmet Reşit Sönmez, Erdal Polat, Mustafa Duman.

**Resources:** Mehmet Reşit Sönmez, Mehmet Ömer Özduman.

**Software:** Mehmet Reşit Sönmez, Gülşah Biçer.

**Supervision:** Mehmet Reşit Sönmez, Ahmet Orhan Sunar, Mehmet Ömer Özduman, Erdal Polat, Mustafa Duman.

**Validation:** Mehmet Reşit Sönmez, Erdal Polat.

**Visualization:** Mehmet Reşit Sönmez, Erdal Polat.

**Writing – original draft:** Mehmet Reşit Sönmez, Ahmet Orhan Sunar.

**Writing – review & editing:** Mehmet Reşit Sönmez, İsa Caner Aydin, Ahmet Orhan Sunar, Erdal Polat, Mustafa Duman.
